# Editor American Journal Dental Science

**Published:** 1906-04

**Authors:** L. A. Sherman

**Affiliations:** Neoga, Ill.


### COMMUNICATIONS.

Editor American Journal Dental Science: In regard to pulp mummification I beg to differ with Dr. Waas' article from *"Items of Interest."*

For more than four years I have been using mummifying paste for root filling and treatment of abscesses to the exclusion of any other preparation, and in all that time I know of only 2 or 3 failures, and I attribute those to the fact that the pulp chamber and canals were imperfectly opened up. No money could tempt me to go back to the old back breaking way of canal filling. Instead of spending the better part of half a day, and nearly causing nervous prostration in my patient, trying to fill impossible canals, and knowing when I got through that in spite of my best efforts, the work was imperfectly done, I open the pulp chamber freely, remove as much of the pulp as possible with a large round bur, fill pulp chamber with the paste, and with a cotton pledget force into the canals until patient feels it, then seal with cement and fill. Time required, fifteen minutes to half an hour. Results, perfect. Except in lower molars where there is a free flow of saliva, I do not even use the dam.

In cases of putrescent pulp and abscess I use the same method except that I force the paste through the foramen if possible. It will cure almost any abscess, and what is more, it will keep it cured. There is usually some soreness of the tooth for a day or two after filling, but that soon disappears, and you can then assure the patient that his troubles with that tooth are over for all time.

I use Gideon Sibley Company's paste.

L. A. Sherman, D. D. S.,

